# *Candida albicans* meningitis in AIDS patient: A case report and literature review

**DOI:** 10.1016/j.idcr.2021.e01216

**Published:** 2021-07-06

**Authors:** K. Bourbeau, S. Gupta, S. Wang

**Affiliations:** Arrowhead Regional Medical Center, Colton, United States

**Keywords:** meningitis, candida, candida meningitis, candida albicans, fungal meningitis, Brain Infection

## Abstract

*Candida albicans* is found to be part of the normal flora in human skin, oral, and respiratory tract, and is known to be an opportunistic infection in immunocompromised populations; rarely is it a cause of meningitis. This case of a patient with Acquired Immune Deficiency Syndrome (AIDS) and *Candida albicans* meningitis illustrates the subtle symptoms and insidious onset of fungal meningitis. This case and review of literature identify the importance of early identification and therapy.

## Introduction

Meningitis presents as a wide range of symptoms, from acute, life threatening bacterial or viral infections, to subacute, subtle fungal infections in immunocompromised populations. Acquired Immune Deficiency Syndrome (AIDS) patients in particular, are even more susceptible to fungal infections due to an impaired cell-mediated immunity.

The incidence of *Candida albicans* meningitis is rare, however, well documented in the literature. In this report, we encounter a rare case of meningitis caused by *Candida albican*s in a young patient with untreated Human Immunodeficiency Virus (HIV) infection. Meningeal infection due to *C. dubliniensis*, *C. glabrata*, and *C.tropicalis* have all been described, yet *C. albicans* remains the leading cause of *Candida* infections [5,6,10]. This case and review of literature demonstrate the subtle clinical findings of fungal meningitis and the importance of the investigative process, early recognition and treatment process of Candida meningitis.

## Case Description

This case involves a 23-year-old female with HIV infection for 6 years, who had been off of highly active antiretroviral therapy (HAART) for 3 years. The patient presented with a headache for 1 month. Associated symptoms included neck pain and stiffness, back pain, fever, nausea, dry cough, as well as a 30 pound unintentional weight loss over the preceding 3 months. Remainder of the review of systems was negative. Physical examination revealed a slender female without evidence of lymphadenopathy, skin rash, cardiorespiratory abnormalities, with temporal wasting and mild nuchal rigidity without other focal findings. Vital signs revealed low grade fever and tachycardia.

Of note, our patient presented to a different ER approximately 1-2 months before for sore throat, at which time she was treated with fluconazole for presumed esophageal candidiasis, and had subsequent resolution of symptoms.

Patient’s workup included negative head computed tomography (CT) and magnetic resonance imaging (MRI) of the brain. Lumbar puncture showed cerebrospinal fluid (CSF) with significant pleocytosis with white blood count (WBC) 997 and hypoglycorrhachia (glucose 24). See Table 1 for remainder of CSF studies. Opening pressure was not reported.

**Table 1:** CSF studies.

	Day 1	Day 7	Day 14
Volume (mL)	6	10	0.5
Appearance	SL-Hazy	clear	cloudy
Color	No color	no color	no color
WBC (/uL)	997	25	14
RBC (/uL)	3	11	31
Neutrophils (%)	72	48	15
Lymphocytes (%)	25	36	30
Macrophages (%)	3	16	55
Glucose (mg/dL)	24	22	N/A
Total Protein (mg/dL)	59	37	N/A
Gram stain	No organisms seen WBC present	No organisms seen	No organisms seen
Culture	*Candida albicans*	*Candida albicans*	No growth
Coccidioides Ab	<1:1		
Cryptococcus Ag	Negative		
West Nile IgG Ab	Negative		
West Nile IgM Ab	Negative		
HSV I DNA Quant PCR	<100		
HSV II DNA Quant	<100		
Haemophilis influenza Ag	Negative		
Neisseria meningitidis Ag	Negative		
Streptococcus pneumonia Ag	Negative		
Streptococcus Group B Ag	Negative		

Blood work and initial CSF studies were negative for the most common bacterial and viral causes of meningitis. Complete blood count was significant for anemia, lymphopenia without leukopenia; comprehensive metabolic panel showed slight hyponatremia without other abnormalities. Serology studies confirmed HIV infection with viremia (4.99 HIV-1 RNA logcopies/ml; 98400 HIV-1 RNA PCR copies/ml), as well as severely decreased CD4 count (<20 cells/uL) (Table 1). Blood cultures were not drawn on this admission.

**Fig. 1:**
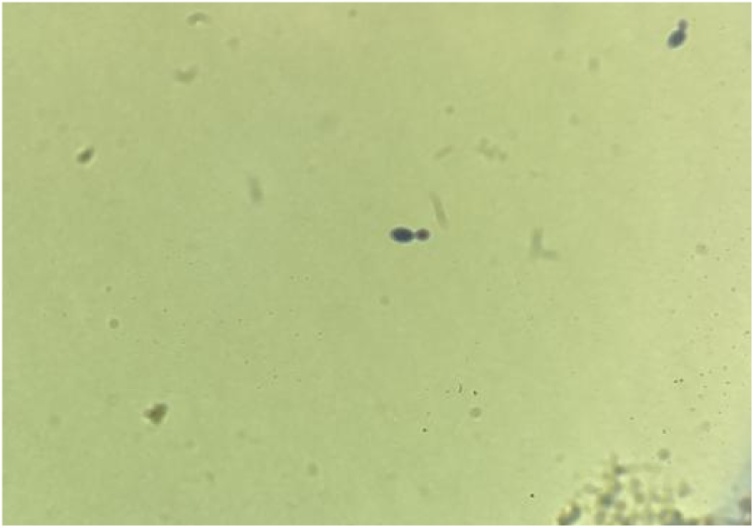
Microscopy of CSF culture demonstrating *Candida albicans*.

Given her presentation and CSF studies, the patient was initiated on empiric vancomycin, ceftriaxone, and acyclovir. However, her symptoms persisted. On day 3 of admission, CSF culture grew *Candida albicans* (Fig. 1). Antibiotics and antiviral treatment were discontinued; she was started on liposomal amphotericin B with oral flucytosine per Infectious Disease consultant’s recommendations. She received weekly lumbar punctures. By day 14, CSF culture was negative, and the patient was transitioned to oral fluconazole consolidation therapy for 4 weeks. Her clinical condition was significantly improved by the day of discharge, with resolution of headache and improvement of CSF pleocytosis. Antiretroviral Therapy (ART) was not restarted during her admission, due to concern for Central Nervous System related immune reconstitution inflammatory syndrome (CNS-related IRIS); plan at time of discharge to initiate one month later in the outpatient setting.

## Discussion

### Risk factors

Candida species is an uncommon etiology for meningitis, but has been described most commonly among immunocompromised populations including HIV/AIDS patients [11], organ transplant recipients [5] cancer patients, and neonatal patients [2,4,6]. Another risk factor includes prior antibiotic use [7]. One rare occurrence was in 2012, when an outbreak of *Candida* meningitis was tied to contaminated Methyl-prednisone among otherwise immunocompetent patients [12]. Amongst *Candida* species, *C. albicans* is most common. Routes of transmission in immunocompetent patients can include hematogenous spread secondary to intravenous drug use [8] and direct inoculation via neurosurgical procedures [3,9]. In several cases, a prior diagnosis of Candida opportunistic infection was established, leading to increased risk for Candida meningitis, as seen with our patient.

### Common signs and symptoms

The clinical presentation of Candida meningitis is nonspecific, but most commonly included headache and fever, in addition to nuchal rigidity, back pain, and fatigue [3,7,9]. Presentations varied from acute to chronic symptoms, mimicking symptoms of more common etiologies of meningitis, including tuberculosis and cryptococcal infection [7]. In neonates, the presentation may be even more subtle, with respiratory compromise, bradycardia, and metabolic acidosis as the indicators of underlying pathology [2]. In other cases, patients presented as suspected bacterial meningitis and treated with empiric therapy; after antibiotic regimens failed to improve symptoms, fungal meningitis was explored further, and antifungal therapy was initiated [8,10]. This was seen in our patient, who had continued symptoms despite initial antibiotics; once positive CSF cultures became positive, treatment was directed accordingly.

### Lab findings

Given the low incidence of *Candida albicans* meningitis, the diagnostic work up relies heavily on history taking in addition to laboratory studies. The gold standard is CSF analysis and culture, although findings are often nonspecific. The most common finding on initial CSF studies is pleocytosis [1,11], seen in our patient, including elevated white cells with neutrophil predominance and hypoglycorrhachia. CSF cultures for fungal species are often of low yield, making a formative diagnosis challenging [5,8]. Patients often undergo multiple lumbar punctures before a definitive diagnosis is made [3,4,10]. In many cases, a presumptive diagnosis was made from blood cultures or extracranial candidiasis, identifying candidemia and correlating with the clinical findings of meningitis [4,11].

### Treatment

The most widely accepted treatment is liposomal amphotericin B, with or without flucytosine [2,3]. Given the rarity of the diagnosis and improvement in the diagnostic work up including improvements in imaging and laboratory techniques, it is no surprise that older case studies described more limited therapeutic options compared to more recent studies. Previously, amphotericin B was used as monotherapy with lower efficacy [2]; other studies among neurosurgical patients described source control alone as treatment with some success [9]. More recent studies recommend what is now the widely accepted standard of combination amphotericin B and flucytosine followed by oral fluconazole [13]. One case study described rescue therapy with voriconazole in the setting of persistent neonatal candidemia [6].

Recommended dosing of initiation intravenous amphotericin B and flucytosine were extrapolated from cryptococcal meningitis with similar dosing and duration. Duration of initial therapy depends on clinical response to therapy and improvement of CSF findings. Patients are then transitioned to maintenance (“step down”) dosing of daily oral fluconazole at 6-12 mg/kg. Recommended duration of maintenance therapy is until clinical signs and symptoms of central nervous system infection are resolved [13].

### Other considerations: Beta-D-glucan

Recent investigators have utilized another marker for fungal infection, beta-D-glucan [5,12]. Beta-D-glucan is a component of fungal cell walls not found in cryptococcus, and can help in diagnosis of fungal meningitis when other studies are nondiagnostic. Some studies describe serum vs CSF levels [8] for diagnosis using various cutoff values. Others have shown as high as 100% sensitivity and 98% specificity, although concerns exist about contamination [12]. Evaluation of CSF levels for beta-D-glucan may prove valuable in the future work up of suspected Candida meningitis in the future, given the difficulty of existing lab techniques and often negative cultures.

## Conclusion

*Candida albicans* is a well-known pathogen in immunocompromised, although uncommon as an etiology of meningitis. A careful work-up including history, physical exam, imaging, and laboratory studies are key in the diagnosis of this rare infection. The patient’s risk factors, including untreated HIV infection with CD4 count <20, recent AIDS defining illness with *C. albicans* esophagitis, when combined with the symptoms of fever, headache, neck and back ache, led the investigating team to the diagnosis.

High index of suspicion and prolonged treatment course with antifungal therapy is recommended until immune reconstitution.

## Author statement

Katherine Bourbeau: Conceptualization, Investigation, Writing – Original Draft; Saloni Gupta: Writing – Review and Editing; Sharon Wang: Supervision, Writing – Review and Editing.

## Funding

No funding was required to complete this work.

## Ethical approval

Written informed consent was obtained from the patient for publication of this case report and accompanying images. A copy of the written consent is available for review by the Editor-in-Chief of this journal on request.

## Consent

Written informed consent was obtained from the patient for publication of this case report and accompanying images. A copy of the written consent is available for review by the Editor-in-Chief of this journal on request.

## Author contribution

Dr. Bourbeau collected data and compiled all tables and charts. She treated the patient as well. She also contributed to writing of the paper. Saloni Gupta, MSIV contributed to the writing and compiling of this paper. Dr. Wang supervised the entire case report and contributed to making edits in the writing. She also was consulted for the case and provided medical advice for treatment.

## Declaration of Competing Interest

The authors report no declarations of interest.
